# Monitoring Drought Tolerance in Oil Palm: Choline Monooxygenase as a Novel Molecular Marker

**DOI:** 10.3390/plants12173089

**Published:** 2023-08-28

**Authors:** Potjamarn Suraninpong, Kannika Thongkhao, Azzreena Mohamad Azzeme, Padungsak Suksa-Ard

**Affiliations:** 1 School of Agricultural Technology and Food Industry, Walailak University, Nakhon Si Thammarat 80161, Thailand; spadungs@wu.ac.th; 2 Biomass and Oil Palm Center of Excellence, Walailak University, Nakhon Si Thammarat 80161, Thailand; 3 School of Languages and General Education, Walailak University, Nakhon Si Thammarat 80161, Thailand; kannika.to@wu.ac.th; 4 Faculty of Biotechnology and Biomolecular Sciences, Universiti Putra Malaysia, Serdang 43400, Selangor, Malaysia; azzreena@upm.edu.my

**Keywords:** drought stress, abiotic stress, SSCP, marker assist selection, full length

## Abstract

Water scarcity negatively impacts oil palm production, necessitating the development of drought-tolerant varieties. This study aimed to develop molecular markers for oil palm breeding programs focused on drought tolerance. Genes associated with drought tolerance were selected, and single nucleotide polymorphism (SNP)-based markers were developed. Genomic DNA was successfully extracted from 17 oil palm varieties, and 20 primers out of 44 were effectively amplified. Screening with single-strand conformation polymorphism (SSCP) revealed an informative SNP marker from the choline monooxygenase (CMO) gene, exhibiting CC, CT, and TT genotypes. Notably, the oil palm variety La Mé showed the CT genotype, while Surat Thani 2 (Deli × La Mé) exhibited the CT and CC genotypes in a 1:1 ratio. Gene expression analysis confirmed the association of the CMO gene with drought tolerance in commercial oil palm varieties. The full-length CMO gene was 1308 bp long and shared sequence similarities with other plant species. However, amino acid sequence variations were observed compared with existing databases. These findings highlight the potential utility of the CMO marker for drought tolerance selection, specifically within the La Mé parent of oil palm Surat Thani 2 varieties, and strongly confirm the La Mé S5 population and Surat Thani 2 as drought-tolerant varieties.

## 1. Introduction

Oil palm (*Elaeis guineensis*) is a crucial plantation crop with significant economic importance in Southeast Asia, Africa, and South America. Renowned for its high productivity in vegetable oil compared with other oil-bearing crops, it serves as a valuable source for products ranging from margarine and cooking oils to animal feeds, soaps, and detergents. In anticipation of its role in green energy in the future, the demand for oil palm has surged, making it a pivotal agricultural commodity in recent years. Given its high productivity, oil palm is known to require relatively substantial amounts of water and humidity. An average annual rainfall of over 2000 mm, evenly distributed throughout the year, is considered ideal for its growth and yield. Consequently, water plays a crucial role in oil palm development. However, drought conditions can directly impact various physiological processes, such as stomatal closure during the day, elevated leaf temperatures leading to decreased photosynthesis rates, and altered reproductive patterns, all of which ultimately contribute to a decrease in oil palm yield [1] (pp. 101–111). In the context of drought stress, oil palm plants respond at cellular, molecular, biochemical, and physiological levels. Drought tolerance, a complex quantitative trait, involves the participation of multiple genes. Advancements in crop physiology and genomics have provided valuable insights into drought tolerance, equipping breeders with the knowledge and tools to improve plant resilience under water-deficient conditions. Hence, enhancing yield under drought stress represents a prominent goal in plant breeding worldwide.

This study aims to monitor molecular markers specific to drought tolerance in commercial oil palm varieties in Thailand, using the single-strand conformation polymorphism (SSCP) technique. SSCP is a widely used method for detecting DNA polymorphisms after amplification with polymerase chain reaction (PCR), especially for identifying point mutations or single nucleotide polymorphisms (SNPs) within a DNA fragment [2]. Its precision, ease of execution in basic laboratory settings, and cost and time efficiency have led to its extensive application in mutation detection and identification across various organisms [3,4,5,6]. The gene of interest, Choline monooxygenase (CMO), belongs to the compatible organic solute group and plays a role in glycine betaine synthesis, a substance produced by plants in response to stress from salinity and drought [7]. Previous studies have reported its involvement in drought and salt tolerance in other plant species, such as spinach, sugar beet, and amaranth [8,9,10]. Although the CMO gene has been extensively studied for its role in drought resistance, its expression in relation to drought tolerance in oil palm has not been previously reported. To assess CMO gene expression under drought stress, the reverse transcription polymerase chain reaction (RT-PCR) method is employed. RT-PCR enables the detection of different levels of expressed mRNA when specific primers designed from a target cDNA sequence are applied. RT-PCR has been widely used in oil palm research to study gene expression, including various genes involved in flower development [11,12], fruit synthesis [13,14], and mantle flowers [15]. Additionally, the full-length sequence of the CMO gene will be determined using the rapid amplification of cDNA ends (RACE) technique. RACE allows for the retrieval of gene sequences from both the 3′ and 5′ ends, facilitating a comprehensive analysis of the gene [16]. The RACE technique, which is rapid and does not require radioactivity, has been previously successful in gene cloning from various organisms [17], including oil palm [15].

In this investigation, we evaluated the effectiveness of molecular markers developed from genes associated with drought tolerance, obtained from both the National Center for Biotechnology Information (NCBI) database and our pyrosequencing database (https://bigd.big.ac.cn/gsa/browse/CRA007757 accessed on 17 August 2020), using the single-strand conformation polymorphism polymerase chain reaction (SSCP-PCR) technique. Furthermore, the CMO gene’s expression will be examined in commercial oil palm varieties under 45 days of drought stress using RT-PCR. The full-length sequence of the CMO gene will be determined via the RACE method, and its evolutionary relationship with other living organisms will be analyzed through a phylogenetic tree comparison.

## 2. Results

### 2.1. Molecular Marker Screening for Oil Palm Drought Tolerance via SSCP

**Figure 1:**
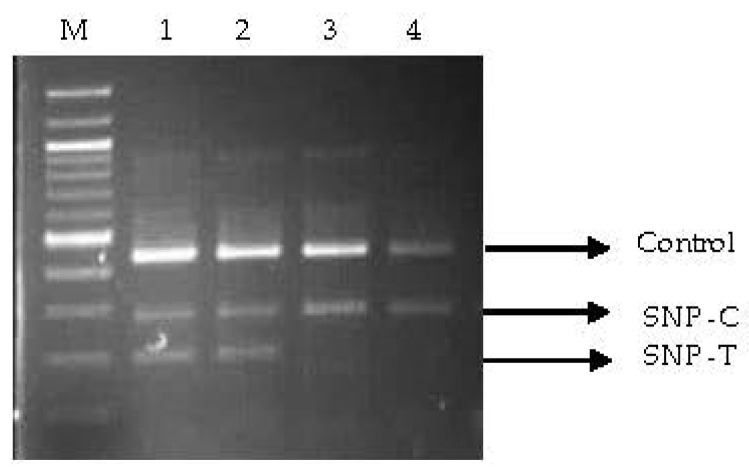
PCR products obtained from CMO1 tetra-primers after amplification with DNA from various oil palm varieties using the SSCP technique. 1 and 2 = La Mè (replications 1 and 2, respectively); 3 = Surat Thani 2; 4 = Univani; M = 100 bp ladder marker.

Out of the 44 primer pairs designed from 12 drought-tolerant genes, 20 primer pairs were successfully amplified across the studied annealing temperature range. However, SSCP monitoring revealed that six primer pairs derived from CMO-03, abscisic acid-04 (ABA-04), monohydroascorbate reductase-04 (MHAR-04), pyrroline-5-carboxylate dehydrogenase-01 (P5CDH-01), helicase-03, and helicase-04 genes exhibited distinguishable DNA banding patterns in oil palm samples (Figure S1). Nucleotide sequences from five out of six primers, namely, CMO-03, ABA-04, MHAR-04, helicase-03, and helicase-04, were analyzed (Figure S2). Each gene exhibited five to nine repeated nucleotide sequences. Analysis of the chromatogram peak sequences identified SNP positions for ABA (one SNP position: T/A), CMO (three SNP positions: C/T, C/T, and T/A), Helicase3 (one SNP position: C/T), helicase4 (one SNP position: C/T), and MHAR (two SNP positions: G/C and T/A) (Figure S3). Among the eight tetra-primers designed from these SNP positions (Table S1), only one tetra-primer, namely, CMO1, yielded a PCR product matching the intended PCR output size. The PCR product sizes for the T, C, and control alleles were 197, 282, and 442 bp, respectively, with an optimum temperature of 50 °C for 30 s (Figure 1).

The examination of the CMO1 tetra-primer using three groups of oil palm samples yielded the following results: In the first group, only 1 out of 17 varieties, La Mé, consistently showed the expected PCR product size for the control gene, C allele, and T allele (CT genotype). Surat Thani 2 exhibited the same PCR product size as La Mé in 20% of the samples. The remaining varieties displayed the PCR product size corresponding to the C allele (CC genotype). In the second group, all samples of La Mé and 40% of the Surat Thani 2 samples, which were subjected to 90 days of dehydration, exhibited the CT genotype, whereas Ghana and Surat Thani 1 displayed the CC genotype. Similar to the third group, all La Mé samples from the farmer plantation demonstrated the CT genotype. Surat Thani 2 exhibited both the CT and CC genotypes in a ratio of 16:9, respectively, while Ghana and Surat Thani 1 exhibited the CC genotype.

### 2.2. CMO Gene Expression under Drought Stress Condition via Real-Time PCR

The RNA extracted from oil palm leaves exhibited a mean content of 217.25 ng/µL and absorbance values ranging from 1.8 to 2.0. The synthesized cDNA, which was validated using the PCR technique with 18S rRNA primers (a housekeeping gene), displayed suitable quality for gene expression studies, resulting in a 530 bp product. The CMO primer, designed based on the nucleotide sequence obtained from the NCBI database (XM_010928966.1), is presented in Table 1. The CMO primer successfully amplified at various temperature ranges, yielding bands of 282 bp. However, an annealing temperature of 60 °C consistently produced specific DNA banding sizes. Therefore, this temperature was selected for further gene expression studies.

**Figure 2:**
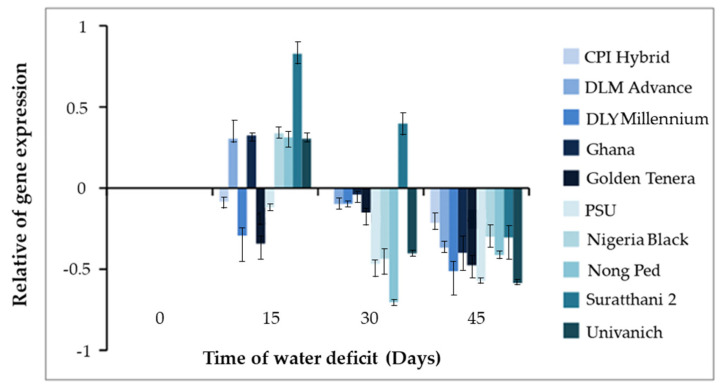
The expression of the CMO gene in various oil palm varieties after 45 days of water deficit.

Under water deficit conditions for 15 and 30 days, Surat Thani 2 exhibited the highest expression of the CMO gene, with values of 0.827 times and 0.397 times, respectively. Nigeria Black Ghana, Nong Ped, Univanich, and DLM Advance followed in terms of expression levels. Notably, Nong Ped displayed the lowest gene expression of −0.703 times after 30 days of water deficit. Following dehydration for 45 days, the expression of the CMO gene decreased in all oil palm varieties. Specifically, Univanich showed the lowest gene expression of −0.584 times, followed by Nong Ped with −0.413 times. Surat Thani 2 exhibited a gene expression of −0.303 times (Figure 2). Unfortunately, none of the oil palm varieties survived beyond 45 days of dehydration, and even with subsequent watering, recovery was not achieved.

### 2.3. Characterization of CMO Full-Length Gene

The RNA extracted from Surat Thani 2 leaves had a concentration of 2350 ng/µL, and the protein ratio A260/A280 was measured as 1.98. The cDNA synthesized from this RNA sample was subsequently amplified using the primers specified in Table 2.

**Figure 3:**
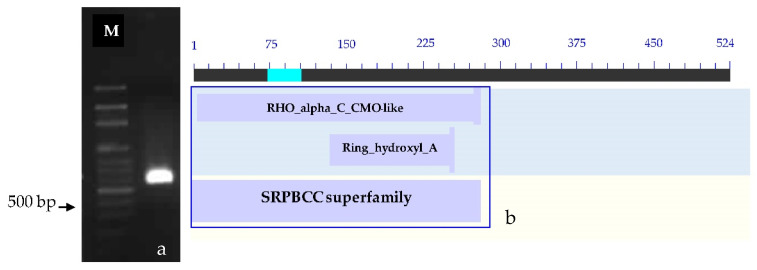
PCR products and comparison of nucleotide sequences at the 3′ end. (**a**) PCR product generated at the 3′ end of the oil palm leaf variety Surat Thani 2. (**b**) Comparison of nucleotide sequences at the 3′ end with the NCBI database. M = 100 bp DNA ladder marker.

The determination of the full-length CMO gene at the 3′ end revealed the presence of sharp DNA fragments, approximately 650–700 bp in size. After cutting, purifying, and sequencing, a portion of the nucleotide sequence measuring 523 bp at the 3′ end was obtained. Alignment analysis of the CMO nucleotide sequence with the NCBI database revealed similarities with both oil palm and date palm. The presence of the two domain positions, RHO_alpha_C_CMO-like and Ring_hydroxyl_A, confirms the complete nucleotide sequence of the CMO gene at the 3′ end (Figure 3).

**Figure 4:**
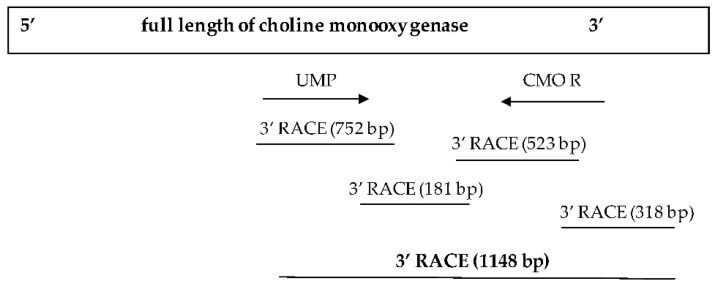
Nucleotide sequence size of the CMO gene at the 3′ end.

**Figure 5:**
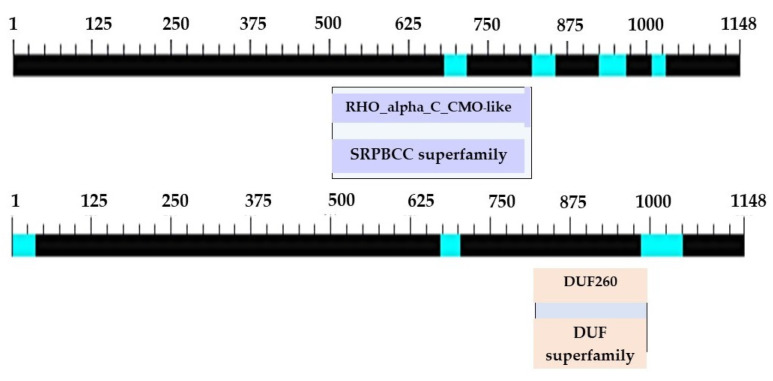
Positions and sizes of the two domains in the complete nucleotide sequence of the PCR product at the 3′ end of the CMO gene from oil palm Surat Thani 2.

The primer was subsequently redesigned to obtain a complete nucleotide sequence at the 3′ end. This time, a sharp DNA banding with fragments approximately 650–700 bp in size was obtained. After cutting, purifying, and determining the nucleotide sequence of the targeted DNA banding, a portion of the obtained sequence from the 3′ side revealed the base sequence that connects the original nucleotide sequence with the newly discovered sequence, measuring 181 bp in size. After completing the 3′ end nucleotide sequence, an additional 318 bp stop codon nucleotide sequence was obtained, resulting in a total CMO nucleotide sequence size of 1148 bp (Figure 4). The obtained nucleotide sequences exhibited similarities to the CMO sequences of oil palm and date palm. Consistent with the earlier findings, two domain positions, RHO_alpha_C_CMO-like and Ring_hydroxyl_A, confirmed the complete sequencing of the CMO gene (Figure 5).

**Figure 6:**
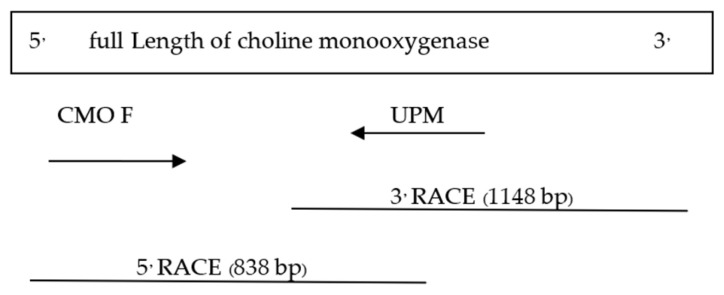
CMO gene nucleotide sequence size at the 5′ end.

**Figure 7:**
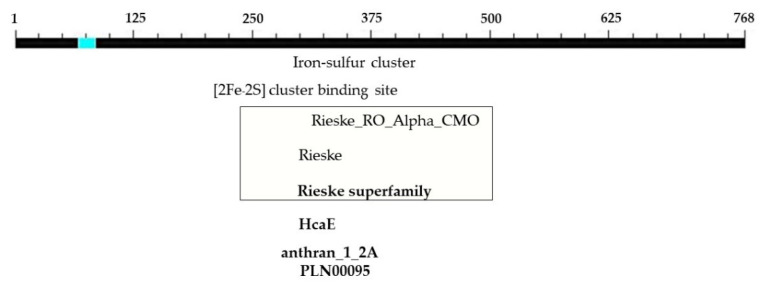
Positions and sizes of five domains in the complete nucleotide sequence of the PCR product at the 5′ end of the CMO gene from oil palm Surat Thani 2.

**Figure 8:**
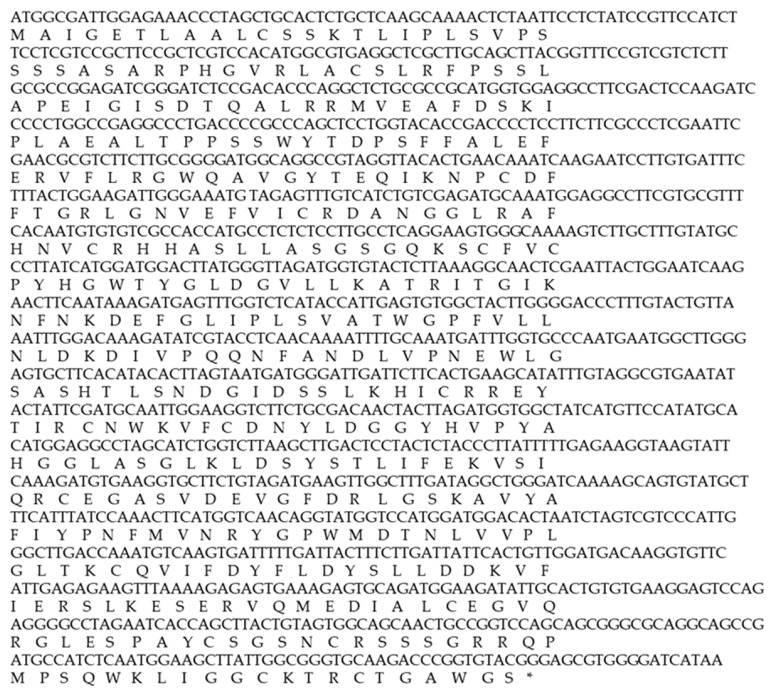
The CMO nucleotide sequence of oil palm variety Surat Thani 2 (* Amino acids translated from nucleotides).

**Figure 9:**
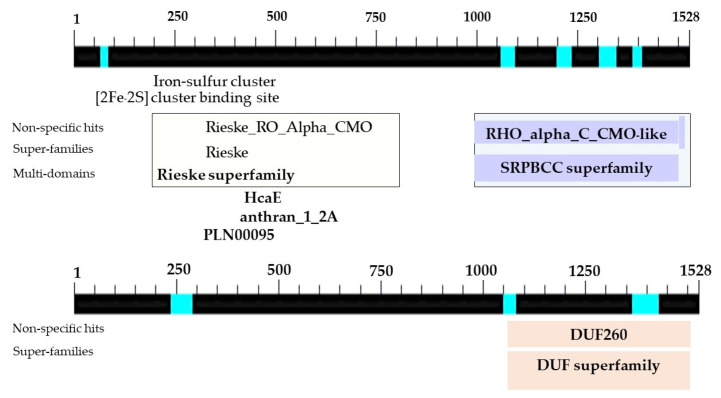
Location and sizes of domains in the nucleotide sequences of PCR products obtained from the CMO complete sequencing of oil palm Surat Thani 2.

The determination of the full-length CMO gene at the 5′ end revealed sharp bands of DNA fragments approximately 1500 bp in size. After cutting, purifying, and sequencing, a portion of the nucleotide sequence at the 5′ end measuring 838 bp was obtained, with the start codon AGT identified (Figure 6). Alignment analysis of the nucleotide sequence with the NCBI database confirmed the similarity of the CMO gene with those of oil palm and date palm. The presence of five domain positions, Rieske_RO_Alpha_CMO, Rieske, HcaE, anthran_1_2_A, and PLN00095, indicated the complete nucleotide sequence of the CMO gene (Figure 7). The cDNA, amplified using the designed primers to generate a complete DNA fragment, resulted in a total length of 1308 bp, with the TAA stop codon observed (Figure 8). The obtained nucleotide sequences exhibited similarities to the CMO gene sequences of oil palm and date palm (Table 3). In total, seven domain positions, including Rieske_RO_Alpha_CMO, RHO_alpha_C_CMO-like, Rieske, HcaE, anthran_1_2_A, PLN00095, and DUF260, confirmed the complete sequencing of the CMO gene (Figure 9).

### 2.4. Evolutionary Correlation of the CMO Gene from Oil Palm Surat Thani 2 with Other Organisms

**Figure 10:**
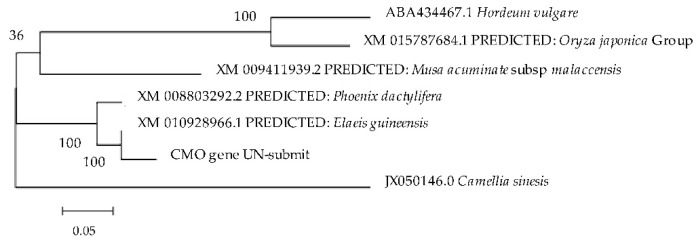
Phylogenetic tree of the CMO gene from Surat Thani 2 in comparison with other organisms.

The evolutionary relationships among the CMO nucleotide sequences were analyzed using phylogenetic trees constructed with the Bootstrap method. Additionally, the genetic relationships between the CMO gene of oil palm Surat Thani 2 and five organisms in the GenBank database (*Camellia sinensis*, *Hordeum vulgare*, *Phoenix dactylifera*, *Oryza sativa* Japonica Group, and *Musa acuminata* subsp. Malaccensis) were investigated using the Kimura two-parameter model and Gamma Distributed (G). The resulting phylogenetic tree revealed three distinct groups. The CMO gene from oil palm Surat Thani 2 belongs to the same group as *P. dactylifera*, followed by *H. vulgare*, *O. sativa* Japonica Group, *M. acuminate,* and *C. sinensis* (Figure 10).

**Figure 11:**
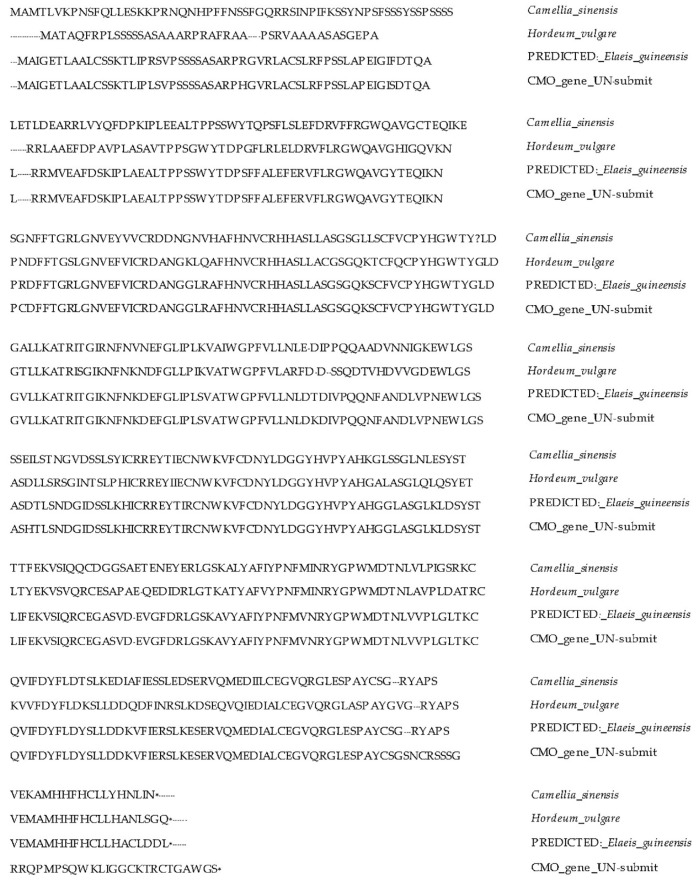
Comparison of CMO nucleotide sequences from Surat Thani 2 with those of other crops (- no amino acid in that position, * the terminal portion of amino acid sequence in each plants).

In terms of comparing the similarity of amino acids in the CMO gene of the Surat Thani 2 oil palm variety with other plants, it was found that the greatest similarity was with oil palm in the NCBI database. However, distinct variations were identified specifically in the coding region’s terminal portion, which was unique to the nucleotide sequence of the Surat Thani 2 oil palm variety but absent in the NCBI and Malaysia Oil Palm databases. These differing parts were the ones where the domain positions were not found when compared with the GenBank database (Figure 11).

## 3. Discussion

Water stress is one of the most common adverse environments in the oil palm life cycle. To counteract with water deficit, most higher plants accumulate compatible solutes, proline, trehalose, late embryogenesis abundant (LEA) protein, reactive oxygen species (ROS), helicase, mannitol, and abscisic acid [18], together with the regulation of stress-related gene expression to adjust their stress [19,20]. Our previously study found change in biochemical induction and gene expression in oil palm drought stress [21]. In this particular study, only the CMO gene designed from the NCBI database was found to exhibit differentiation among the oil palm drought varieties. This suggests that there are genetic variability and distinct genetic variations specifically associated with the drought response in this gene. The CMO gene may possess unique markers or mutations that enable effective differentiation among the studied varieties. Its involvement in the relevant biochemical pathways and its sensitivity to different drought conditions contribute to the observed differences. It is possible that the other genes investigated in the study did not capture the necessary genetic variations or were less responsive to drought conditions [22,23,24]. It is worth noting that the SSCP technique used in this study has limitations in detecting gene variations, which could have influenced the detection process [25]. Further research employing advanced techniques is recommended to explore a broader range of genetic variations associated with drought response.

However, the SSCP technique successfully distinguished the C/T SNP position from the CMO-03 nucleotide sequence of the CMO gene, revealing small and distinct DNA fragments of T, C, and the control allele at sizes of 197, 282, and 442 bp, respectively. This distinction was particularly prominent in the oil palm variety La Mé, an S5 parent of Surat Thani 2. SSCP markers are highly sensitive and effective research tools when the DNA fragment size is small, ranging from 100 bp to 400 bp [26] or 200 to 600 bp [27]. SSCP is PCR-based, polymorphic, and co-dominant, similar to microsatellites. However, SNPs occur more frequently than microsatellites, making them easier to develop as molecular marker [28]. PCR-SSCP markers have an advantage in determining homozygote and heterozygote genotypes, as well as the inheritance pattern of alleles. This enables the accurate identification of drought tolerance characteristics in different oil palm varieties. However, this protocol requires specific conditions such as running time and temperature, applied power, gel concentration, and thickness [27]. In this study, the main factor affecting the non-amplified PCR product was the size of the tetra-primer and the annealing temperature. The presence of the SNP obtained from CMO genes in oil palm, which has not been previously reported, suggests that CMO genes related to drought tolerance have been studied in other crops such as rice [29], maize, and sorghum [30], as well as cotton [31]. The tetra-primers designed from CMO-03 revealed CT genotypes in La Mé and exhibited CT and CC genotypes in Surat Thani 2, the cross between Deli and La Mé. CC refers to a homozygous genotype that has two identical copies of the “C” allele at that specific SNP location, whereas CT refers to a heterozygous genotype that has one “C” allele and one “T” allele at that specific SNP location. These genotype designations are often used in genetic studies to identify variations that may be associated with certain traits or diseases [32]. Thus, the occurrence of CC and CT genotypes confirms the genetic distribution of CMO genes from parents to their hybrids. On the contrary, the marker CMO-03 did not detect CT genotypes in the oil palm varieties DLM Advance and DLY Millennium, which had La Mé as a parent. This result occurred because the La Mé used in this study was an S5 population, a selfing parent from Suratthani Oil Palm Research Center, Thailand. Thus, La Mé genetic differentiation resulted in the CMO-03 marker being undetectable. For confirmation of CMO gene expression, the oil palm varieties were examined under water deficit conditions.

Further, the highest expression of the CMO gene was observed after 15 and 30 days of dehydration, followed by a reduction in expression after 30 days of dehydration. These findings provide stronger evidence for the CMO gene as a candidate gene involved in the drought tolerance response in the oil palm variety Surat Thani 2. The above obtained result was in correlation with the physiological responses of all oil palm varieties under dehydrated conditions. The oil palm Surat Thani 2 showed the highest values for radius canopy, leaf area, and chlorophyll content, compared with the other oil palm varieties. Meanwhile, the oil palm varieties DLM Advance and DIY Millennium showed the highest stomata number, stomata size, and guard cell size compared with the other varieties (unpublished data). This finding is also consistent with studies on CMO gene expression in sugar beet and amaranth [10], *Amaranthus tricolor* [7], and *Beta vulgaris* [33] under dehydration conditions, which showed an increase in CMO gene expression in response to glycine betaine (GB) synthesis. The CMO gene is responsible for synthesizing glycine betaine, an osmoprotectant, which plays a crucial role in the osmotic stress response in higher plants. GB, an effective compatible solute, helps stabilize membranes, protect proteins and photosystem II, and mitigate oxidative damage [33]. It is synthesized through a two-step oxidation process from choline and betaine aldehyde, catalyzed by choline monooxygenase (CMO) and betaine aldehyde dehydrogenase (BADH), respectively [8]. GB stabilizes membranes, protects proteins and photosystem II, and mitigates oxidative damage [34] while the plant is under dehydrated stress or prevents cell damage when exposed to stress conditions such as drought, salinity, and low-temperature environments [35,36,37,38]. Similar to spinach, the CMO gene in oil palm is likely involved in glycine betaine biosynthesis and stress response. However, the specific characteristics and regulatory elements of the oil palm CMO gene remain to be elucidated. Notably, CMO is unique to plant species and is absent in animals and bacteria [9]. This absence of CMO in rice contributes to the lack of glycine betaine accumulation in this crop. Both CMO and the BADH enzyme, which are targeted to chloroplasts, show increased activity during salt stress [39]. Overall, our findings highlight the limited response of CMO to drought stress in oil palm and emphasize the need for further research to understand its role and regulation in this particular plant species. However, the presence of the CMO genotype in this study strongly supports our previous research, indicating that La Mé and Surat Thani 2 are drought-tolerant varieties [21]. Furthermore, we examined the total nucleotide sequence of the CMO cDNA gene in Surat Thani 2, which was found to be 1308 bp in length and contained seven transcribed positions encoding amino acids. The Rieske_RO_Alpha_CMO, RHO_alpha_C_CMO-like, and Rieske domains are involved in the CMO gene synthesis process, which is initially triggered for the synthesis of glycine betaine through the production of choline. Choline is a protein with multiple components that functions in various oxidation reactions, enabling the CMO gene to perform its tasks [40]. HcaE is a part of the 3-phenylpropionate dioxygenase that acts as a transporter of ions and facilitates the acceleration of metabolism [41]. Anthran_1_2_A is an intermediate in the synthesis and degradation of tryptophan, converting anthranilate into catechol, which plays a role in energy metabolism, amino acid synthesis, and amines formation [42], whereas PLN00095 has not been clearly reported yet but is speculated to be a chlorophyllide a oxygenase involved in the synthesis of chlorophyllide b. As for DUF260, it is a protein with an unknown functional role, whereas PLN00095 and DUF260 have not been clearly reported yet, but it is speculated that PLN00095 may be a chlorophyllide a oxygenase involved in the synthesis of chlorophyllide b, while DUF260 is a protein with an unknown functional role. These transcribed positions are crucial for the synthesis of glycine betaine through the production of choline, involving a multicomponent protein that participates in various oxidation reactions [40,41,42,43].

Moreover, the study on the evolutionary relationship of all nucleotide sequences of the CMO gene in the Surat Thani 2 oil palm variety showed a high similarity to oil palm sequences in the NCBI database. However, when examining the amino acid sequences, slight differences were observed in the terminal coding region, which does not contain any domain positions. These findings suggest that there are no significant functional differences in the CMO gene when compared with other plants. The effects of amino acid substitutions on gene structure and function can be diverse, impacting various aspects such as protein folding, stability, enzymatic activity, and protein–protein interactions [41,42,43,44]. Regarding the CMO-03 gene, the change from serine to phenylalanine does not appear to result in significant modifications to the protein’s structure and functional properties. As drought tolerance is a complex trait influenced by multiple genetic factors, with genes involved in drought tolerance playing crucial roles in stress signaling, osmotic regulation, water transport, and other mechanisms related to maintaining cellular homeostasis under water-deficient conditions [44,45]. The altered amino acid sequence of the CMO-03 gene may be a special characteristic of the Surat Thani 2 or La Mé varieties that contributes to the regulation of drought tolerance-related processes in plants. However, further investigations are needed to determine the precise functions of the CMO-03 gene in oil palm or other higher plants. Thus, conducting additional analyses and investigations can provide valuable insights into the molecular mechanisms underlying the involvement of the CMO-03 gene in drought tolerance and its potential implications for plant adaptation to water-limited environments.

## 4. Materials and Methods

### 4.1. Molecular Marker Screening of Oil Palm Drought Tolerance via SSCP

#### 4.1.1. DNA Extraction

DNA was extracted from the leaves of all ten oil palm varieties, as described in Table 4, using a modified version of the Doyle and Doyle [46] method. The oil palm varieties were 20–30 years old. Young oil palm leaves were cut into approximately 5 g pieces and crushed in liquid nitrogen, and 1000 μL of CTAB extraction buffer (20 mM EDTA pH 8.0, 100 mM Tris-HCl pH 8.0, 2% CTAB, 2% PVP-40, 1.4 M NaCl, and 2% β-mercaptoethanol) was added. The mixture was incubated at 60 °C for 1–2 h, followed by the addition of 500 μL chloroform. The mixture was then centrifuged at 12,000 rpm at 4 °C for 2 min (5804R Eppendorf Centrifuge #3591396, Eppendorf, Hamburg, Germany). The supernatant (approximately 700 µL) was transferred to a new 1.5 mL microtube, and 5 µL of RNase was added. The mixture was incubated at 37 °C for 30 min, followed by the addition of 700 µL isopropanol. After gentle shaking to allow DNA precipitation, the mixture was centrifuged at 12,000 rpm at 4 °C for 10 min. The DNA pellet was washed with 1000 μL 70% ethanol, dried at 55 °C, and dissolved in 50 μL TE buffer (10 mM Tris-HCl pH 8.0 and 1 mM EDTA pH 8.0). The extracted DNA was stored at −20 °C until further use.

#### 4.1.2. Primer Design

All CMO nucleotide sequence data from the NCBI database were collected in FASTA format for subsequent alignment and primer design. The ClustalX 1.81 multiple alignment program (http://en.kbdown.com/3000.html accessed on 30 July 2022) was used for the alignment of these sequences to identify suitable positions for primer design. However, genes related to drought tolerance were selected from our pyrosequencing database for primer design. Eleven genes of interest were identified, including proline-related genes (glutathione reductase; GR, arginase; Arg and P5CDH), glycine betaine-related genes (BADH), antioxidant-related genes (superoxide dismutase: SOD, ascorbate peroxidase; APX, MHAR and catalase gene), a trehalose-related gene (TRE), a helicase gene (Helicase), and an abscisic acid gene (ABA). The primer was designed as 18–23 bp with the PCR product size of 200–300 bp, 40–60% CG content, 50–70 °C salt adjusted with both forward (F) and reverse (R). The primer design was performed using the Primer3 Input tool (http://bioinfo.ut.ee/primer30.4.0/, accessed on 11 March 2021) and the Oligonucleotide Properties Calculator (http://www.basic.northwesternedu/biotools/oligcalc.html, accessed on 11 March 2021).

#### 4.1.3. Single Strand Conformation Polymorphism (SSCP)

At the initial stage of the SSCP method, DNA samples from the oil palm variety Surat Thani 2 were analyzed. The primer selection was performed via PCR conditions (T100TM Thermal Cycler, BIO-RAD, Hercules, CA, USA) as described in Table 5. In a 10 μL PCR reaction, 2 μL of DNA template, 5 μL of Toptaq buffer (Qiagen, Hilden, Germany), 0.25 μL of forward primer, 0.25 μL of reverse primer, and 2.5 μL of sterile dH2O were mixed. Subsequently, the obtained primers were used to amplify DNA from various oil palm varieties. The amplified PCR solutions were heated at 95 °C for 10 min and rapidly cooled on ice, and 5 μL of the solution was loaded onto an 8% non-denaturing polyacrylamide gel (29:1) (5%). Electrophoresis was performed in 0.5× TBE buffer at 4 °C for 12 h. The DNA bands were visualized by staining with silver nitrate. Distinct DNA bands with different conformations were observed on the gel and were excised for further analysis. The DNA bands were purified using the QIAquick PCR Purification kit (Qiagen, Hilden, Germany), according to the manufacturer’s instructions. The purified DNA fragments were then subjected to a second round of PCR amplification using the same gene-specific primers. The resulting PCR products were sequenced to obtain the nucleotide sequences. These sequences were aligned with the NCBI database using BLASTN to compare their similarity with known sequences from other crop plants and identify potential SNPs.

To validate the identified SNPs, a tetra-primer was designed by introducing a mismatch base prior to the SNP position. Additionally, a control primer covering the nucleotide sequence at the SNP position was designed for use as a control. To further verify the designed primers, cDNA samples from oil palm varieties that contained the SNP sequence position were used. The PCR conditions for SNP validation are described in Table 5. The PCR reaction mixture for this step contained 1 μL of DNA template, 0.25 μL of control-forward primer, 0.75 μL of SNP reverse primer, 0.75 μL of SNP forward primer, 0.25 μL of control-reverse primer, 5.0 μL of Toptaq, and 2 μL of sterile dH2O. These PCR conditions were applied to three groups of oil palm samples: (1) 17 varieties from The Oil Palm Research Center (Surat Thani Province, Thailand) and private companies in Thailand, with four replications of each variety; (2) four dehydrated oil palm varieties for 90 days, with five replications per variety; and (3) four oil palm varieties from farmer plantations in Surat Thani Province and Chumphon Province, Thailand, with five replications.

### 4.2. CMO Gene Expression under Drought Stress Condition via Real-Time PCR

#### 4.2.1. Oil Palm Drought Stress Condition

Ten oil palm varieties as mentioned in Table 4, at 11 months old, were grown in 36-inch plastic pots filled with a composite soil mixture in a greenhouse at Walailak University, Thailand. Ten plants were grown for each variety. The greenhouse was maintained at a temperature of 28 ± 2 °C and 80% relative humidity. Water stress was conducted by well-watering oil palm plants until the tensiometer reached 0 centibar, and then irrigation was stopped for 45 days. Conversely, for the control group, well-watering was performed to maintain the tensiometer at 45 centibar for the entire 45 days. In this experiment, each of the five oil palm plants was used for both the irrigation and no irrigation treatments. Leaf samples were collected from the second leaf stalk of all oil palm plants every 15 days after water treatment and stored in liquid nitrogen at −80 °C for further analysis.

#### 4.2.2. RNA Extraction

RNA extraction from crushed oil palm leaf samples (0.5 mg) was carried out using the Total RNA Mini Kit following the manufacturer’s instructions (Promega, Madison, WI, USA). The extracted RNA samples were then evaluated for quality and quantity using agarose gel electrophoresis to assess RNA integrity, a NanoDrop spectrophotometer (NanoDrop™ 2000/2000c Spectrophotometers, ND2000CLAPTOP, Thermo Scientific™, Waltham, MA, USA) for measuring RNA concentration, and PCR analysis with the 18S rRNA reference gene to ensure successful RNA extraction and integrity.

#### 4.2.3. cDNA Synthesis

The RNA extracted from the oil palm leaf samples was reverse transcribed into cDNA using the Transcriptor High Fidelity cDNA synthesis kit (Merck, Darmstadt, Germany). The cDNA synthesis reaction was performed by incubating the mixture at 65 °C for 10 min, followed by cooling on ice. Subsequently, the reaction was incubated at 45 °C for 30 min, 85 °C for 5 min, and 95 °C for 5 min. The reaction mixture (20 µL) consisted of 2.0 µL total RNA (2 µg), 1.0 µL oligo (dT) primer (2.5 µM), 8.5 µL H2O, 4.0 µL reaction buffer (5 times), 0.5 µL protector RNase inhibitor (40 units/µL), 2.0 µL deoxynucleotide mix (dNTP), 1.0 µL reverse transcriptase, and 1.0 µL DTT. The efficiency of the cDNA synthesis was verified by performing PCR using 18S rRNA primers. The PCR conditions were as described in Table 5. The resulting PCR products were stored at 4 °C for subsequent gene expression analysis.

#### 4.2.4. Gene Expression Analysis

The nucleotide sequence of the CMO gene obtained from NCBI was used for primer design with the conditions mentioned in Section 4.1.2. For gene expression analysis, a real-time PCR approach was employed using a 7300 Real-time PCR system (Applied Biosystems, Foster City, CA, USA) with EvaGreen® Super mix (BioRad, Hercules, CA, USA). The 10 µL PCR reaction mixture consisted of 1 µL cDNA (diluted 10 times), 5 µL Toptaq, 0.5 µL oligo (dT) primer (1.0 µM), 0.5 µL arbitrary primer (1.0 µM), and 3.0 µL dH2O. The cDNA samples from various oil palm varieties at different dehydration time points (0, 15, 30, and 45 days) were used as templates. Forward and reverse primers specific to the target gene were used for PCR amplification. The PCR conditions were described in Table 5. Three replicates were performed for each sample, and the expression levels were normalized to the 18S rRNA reference gene. Standard DNA at various concentrations (1, 10−1, 10−2, 10−3, and 10−4) was used for comparison. The gene expression results were analyzed using the 2−ΔΔCT method [48].

### 4.3. Characterization of CMO Full-Length Gene and Evolutionary Correlation Study

#### 4.3.1. RNA Extraction

RNA extraction was performed using a modified version of Corre et al.’s [49] method. Oil palm leaf samples from Surat Thani 2 (Deli × La Mé) weighing 1.5–2 g were crushed in liquid nitrogen and mixed with 25 mL of extraction buffer. The extraction buffer consisted of three solutions: solution A (containing guanidium isothiocyanate, 3 M sodium acetate, and 0.1% DEPC), solution B (containing 0.1% sarcosine and DEPC), and solution C (containing 0.1% polyvinylpyrrolidine and DEPC). The mixture was vortexed and placed on ice for 10 min. After centrifugation at 4000 rpm for 40 min, the supernatant was transferred to a new tube and allowed to reach room temperature. Cesium chloride was added, and the mixture was centrifuged at 25,000 rpm at 20 °C for 20 h. The RNA sediment was washed with 70% ethanol, dried, and dissolved in RNase-free water. The quality and quantity of RNA were assessed using a NanoDrop spectrophotometer and agarose gel electrophoresis.

#### 4.3.2. Identification of CMO Full-Length Gene

mRNA was isolated from total RNA using PolyATtract® mRNA Isolation System I and II (Promega, Madison, WI, USA) as per the manufacturer’s instructions. The isolated mRNA was then converted to cDNA using SMARTScribe Reverse Transcriptase enzyme and the Smarter® RACE 5′/3′ kit (Takara Bio, San Jose, CA, USA). In a 20 µL reaction, 1.0 µL mRNA (1 µg), 1.0 µL 3′ RACE Coding Sequence (CDS) primer A (12 µM) or 5′ RACE CDS primer A (12 µM), 10.0 µL H2O, 4.0 µL First-Strand buffer (5 times), 0.5 µL DTT (100 µM), 1.0 µL dNTPs (20 µM), 0.5 µL RNase Inhibitor (40 units/µL), and 2.0 µL SMARTScribe Reverse Transcriptase were mixed. The reaction was incubated at 42 °C for 90 min, followed by incubation at 70 °C for 10 min.

The nested PCR primer design for the amplification of DNA fragments involved using gene-specific primers (GSPs) based on the analytical results of the 3′ and 5′ ends of the CMO gene. The primers were designed using Primer3 Input (version 0.4.0) and the Oligonucleotide Properties Calculator. They were designed to target a region within 100–200 nucleotides of the CMO gene sequence, with a length of approximately 18–25 base pairs, a GC content of 40–60%, and a melting temperature (Tm) adjusted to 50–70 °C. The nested PCR primers consisted of outer gene-specific primers and inner gene-specific primers. The inner primer was designed to be specific and precise in binding to the DNA template strands before synthesis, ensuring no overlap with the base sequences of the outer primer.

The cDNA obtained from the oil palm samples of the Surat Thani 2 variety was amplified to obtain the nucleotide sequences at the 3′ and 5′ ends of the CMO gene. For the amplification of the 3′ end, the outer gene-specific primer (GSP-CMO1) was used, while for the 5′ end, GSPs and the Universal Primer Mismatch (UPM) primer were used. Each PCR reaction had a total volume of 25 µL per sample, consisting of 1.25 µL cDNA (10× dilution), 0.50 µL GSP-CMO1 primer (for the 3′ end) and/or GSPs (for the 5′ end), 2.50 µL UPM primer, 12.50 µL SeqAmp Buffer, 0.50 µL SeqAmp DNA Polymerase, and 7.75 µL dH2O. The PCR conditions included an initial denaturation at 94 °C for 3 min, followed by 35 cycles of denaturation at 94 °C for 30 s, annealing at 58 °C for 30 s, and extension at 72 °C for 3 min. The reaction was then incubated at 72 °C for 10 min and held at 4 °C before analyzing the PCR products using agarose gel electrophoresis. The obtained PCR product from the first amplification step was then diluted 10 times and used as a template for the second amplification step. For the 3′ end, the inner gene-specific primer (GSP-CMO2) was used, while for the 5′ end, GSPs were used along with the universal primer. The PCR reaction volume of 25.00 µL per sample included 5.00 µL (10× dilution) of the PCR product from the first stage, 1.00 µL GSP-CMO2 primer (for the 3′ end) or GSPs (for the 5′ end), 1.00 µL universal primer, 12.50 µL SeqAmp Buffer, 0.50 µL SeqAmp DNA Polymerase, and 5.00 µL dH2O. The PCR conditions for the second stage consisted of an initial denaturation at 94 °C for 30 s, followed by 20 cycles of denaturation at 94 °C for 30 s, annealing at 65 °C for 30 s, and extension at 72 °C for 3 min. The reaction was then incubated at 72 °C for 10 min and held at 4 °C before analyzing the PCR products using agarose gel electrophoresis.

Based on the results of DNA band testing using agarose gel electrophoresis, the DNA strips were cut from the gel for purification using a PCR clean-up and gel extraction kit (Smarter RACE 5′/3′ kit). The cut DNA bands were weighed, placed in 1.5 mL tubes, and added to NTI buffer in a quantity twice the weight of the DNA bands. The mixture was then incubated at 50 °C for 10 min to dissolve the agarose gel. The resulting sample was transferred to a clean-up column and centrifuged at 11,000 rpm for 30 s to immobilize the DNA on the filter. The supernatant was removed, and the DNA was washed twice with 700 µL of wash buffer (NT3) by centrifuging at 11,000 rpm for 30 s each time. The columns were then placed in 1.5 mL Eppendorf tubes, and 50 µL of elution buffer (NE) was added to each tube. After centrifuging at 11,000 rpm for 1 min, the resulting DNA was sequenced to determine the nucleotide sequence. The sequences were subsequently analyzed for completeness and aligned with the NCBI database.

### 4.4. Evolutionary Correlation Study

The nucleotide sequence of the CMO gene obtained from our transcriptome analysis of oil palm drought characteristics was used to design primers using the method and primer design conditions mentioned earlier. The designed primers were then used to amplify the cDNA samples with the following PCR conditions: an initial denaturation at 95 °C for 3 min for 1 cycle, followed by 35 cycles of denaturation at 95 °C for 30 s, annealing at 50–65 °C for 30 s, and extension at 72 °C for 1 min. The reaction was further incubated at 72 °C for 7 min and held at 4 °C before analyzing the PCR products using agarose gel electrophoresis. The PCR reaction had a total volume of 10 µL per sample, consisting of 2.0 µL DNA (10× dilution), 0.25 µL forward primer, 0.25 µL reverse primer, 5.0 µL Toptaq polymerase, and 2.5 µL dH2O. After examining the PCR products on agarose gel, the DNA bands were cut from the gel and purified using a PCR clean-up and gel extraction kit (Smarter RACE 5′/3′ kit). The purified DNA samples were then sent for sequencing. The obtained nucleotide sequences were analyzed for complete sequencing and aligned with the NCBI database to confirm their identity. To explore the evolutionary correlation and similarity of the CMO genes from our oil palm variety with other living organisms, a phylogenetic tree analysis was performed. This analysis allows us to assess the evolutionary relationships and genetic similarities between different CMO gene sequences from various organisms.

## 5. Conclusions

Water stress presents a significant challenge for oil palm throughout its life cycle. To adapt and survive under such conditions, oil palm exhibits mechanisms of drought tolerance, including the accumulation of compatible solutes and the regulation of stress-related genes. In this study, molecular markers were employed using the SSCP technique. Notably, we successfully identified a C/T SNP position in the CMO-03 gene, enabling differentiation among oil palm genotypes, with particular significance in the La Mé S5 family. Furthermore, the discovery of the full-length CMO gene strongly suggests that Surat Thani 2 and La Mé are oil palm drought-tolerant varieties. However, further research is needed to fully comprehend the precise role of the oil palm CMO gene and its implications for oil palm’s adaptation to water-limited environments. Based on our findings, this study enhances our understanding of drought tolerance in oil palm and provides valuable insights for future breeding efforts that utilize molecular markers.

## Figures and Tables

**Table 1:** Primer designed for use in the CMO gene expression analysis.

Primer	Forward Primer (5′ ≥ 3′)	Reverse Primer (5′ ≥ 3′)	PCR Product (Base Pair)
CMO-03	CCT CCC AAA TTA TGT GGT TC	TGC TTT TGA TCC CAG CCT AT	282

**Table 2:** Primer designed for identifying the nucleotide sequence at the 3′ end and the 5′ end of the CMO gene.

Primer Name	Nucleotide Sequence
3′ forward primer (F: 5′ ---> 3′)	
CMO1	GAG AAG GTA AGT ATT CAA AGA TGT G
CMO2	GGC TGG GAT CAA AAG CAG TGT ATG C
5′ reverse primer (5′ ---> 3′)	
CMO3	GAT CCC AGC CTA TCA AAG CCA AC
CMO4	GGT CAA GCC CAA TGG GAC GAC TAG
CMO5	GAA GAC CGT TAG GTC CGT TAC A
CMO6	CCT CTT CAG ATT CTA TAG CCG C
CMO7	CCC AGC CTA TCA AAG CCA ACT TC

CMO: choline monooxygenase.

**Table 3:** BLASTN results of the CMO nucleotide sequence with the NCBI database.

Similarity	Accession No.	E Value	Similarity (%)
Predicted: *Elaeis guineensis* choline monooxygenase, chloroplastic (LOC105049342), mRNA	XM_010928966.1	0.0	99
Predicted: *Phoenix dactylifera* choline monooxygenase, chloroplastic (LOC103715608), mRNA	XM_008803292.2	0.0	94

**Table 4:** Characteristic of various oil palm varieties used in this study.

Variety	Parent	Yield (Ton/0.16 hectare/year)	% Oil	Foliar Length (m)	Average Bunch Weight (kg)	Average Height/Year (cm)	Drought Tolerance	Countries
CPI Hybrid	Chumphon Dura × Chumphon Pisifera	5.0–6.0	>25.0	6.0–7.0	>22.0	40.0	Tolerance	Thailand
Ghana (Themba)	Deli × Ghana	5.45	27.5	8.0	21.3	58.0	Moderate tolerance	Costa Rica
La Mé (S1 parent)	Deli × La Mé	4.89	25.5	9.0	21.4	53.0	Moderate tolerance	Thailand
DLM Advance	Deli × La Mé	5.0	26–28	4.1–6.5	>22.0	36.0–40.0	Tolerance	Africa
DLY Millennium	Deli × La Mé	5.0	24–26	4.3–6.5	>22.0	36.0–40.0	Tolerance	Africa
Golden Tenera (KB)	Ulu Remis Dura and Banting Dura × SP540 and Dumy AVROS	4.5–5.0	26–30	7.5–8.0	>22.0	50.0	Tolerance	Thailand
PSU	Deli × AVROS	>4.0	27	7.0–8.0	21.4	35.0–40.0	Tolerance	Thailand
Nigeria Black	Deli × Nigeria black		30	7.6–8.0	>22.0	50.0–55.0	Moderate tolerance	Costa Rica
Nong Ped	Deli × Yangambi from Malasia	5.3–6.5	28–30	6.0–6.5	>22.0	30.0	Moderate	Malaysia
Univanich	Deli × Yangambi	4.0	24–29	6.0–8.0	>22.0	50.0–60.0	Moderate	Thailand
Suratthani 2	Deli × La Mé	3.6–5.0	23	4.3	17.4	48.0	Tolerance	Thailand

Department of Agriculture [47].

**Table 5:** PCR conditions in each step used in this study.

Step	PCR Condition	No. of Cycles	No. of Replications
4.1.3 primer selection	94 °C for 3 s, 94 °C for 30 s, 40–60 °C for 30 s, 72 °C for 60 s, and 72 °C for 7 min, and cooling at 4 °C.	40 cycles	2
4.1.3 SNPs validate	94 °C for 3 min, 94 °C for 30 s, 50 °C for 30 s, 72 °C for 1 min, 72 °C for 7 min, and cooling at 4 °C.	40 cycles	4
4.2.3 cDNA verified	95 °C for 4 min, 95 °C for 30 s, 55 °C for 30 s, 75 °C for 1 min, and cooling at 4 °C	35 cycles	2
4.2.4 gene expression analysis	95 °C for 2 min, 95 °C for 30 s, 60 °C for 30 s, 72 °C for 1 min, and cooling at 4°C.	40 cycles	3

All PCR products were visualized on a 1.0% agarose gel stained with ethidium bromide, except for gene expression analysis.

## Data Availability

Data are contained within the article.

## Supplementary Materials

The following supporting information can be downloaded at https://www.mdpi.com/article/10.3390/plants12173089/s1: Figure S1: DNA fingerprints of oil palm sample examined with difference gene primers via SSCP in 29:1 nondenaturing polyacrylamide gel; Figure S2: Nucleotide sequence of difference primers; Figure S3: Chromatogram sequence revealed SNP positions of various genes; Table S1: Nucleotide sequence of primers used for molecular marker screening; Table S2: Tetra primer of various genes designed from the SNP positions.

## Abbreviations

ABA = abscisic acid; APX = ascorbate peroxidase; Arg = arginase; BADH = betaine aldehyde dehydrogenase; CDS = coding sequence; CMO = choline monooxygenase; GB = glycine betaine; GR = glutathione reductase; GSPs = gene-specific primers; LEA = late embryogenesis abundant protein; MHAR = monohydroascorbate reductase; NCBI = National Center for Biotechnology Information; P5CDH = pyrroline-5-carboxylate dehydrogenase; PCR = polymerase chain reaction; RACE = rapid amplification of cDNA ends; ROS = reactive oxygen species; RT-PCR = reverse transcription polymerase chain reaction, SSCP = single-strand conformation polymorphism; SSCP-PCR = single-strand conformation polymorphism-polymerase chain reaction; SNP = single nucleotide polymorphism; SOD = superoxide dismutase; TRE = trehalose-related gene; UPM primer = Universal Primer Mismatch.
