# Supplementary material for: Monitoring Drought Tolerance in Oil Palm: Choline Monooxygenase as a Novel Molecular Marker
Source: Plants (Basel). 2023 Aug 28;12(17):3089. doi: 10.3390/plants12173089 (PMC10490023; doi:10.3390/plants12173089)
Supplement: Supplementary file 1 [file plants-12-03089-s001.zip › plants-2529663-Supplementary Materials.pdf]

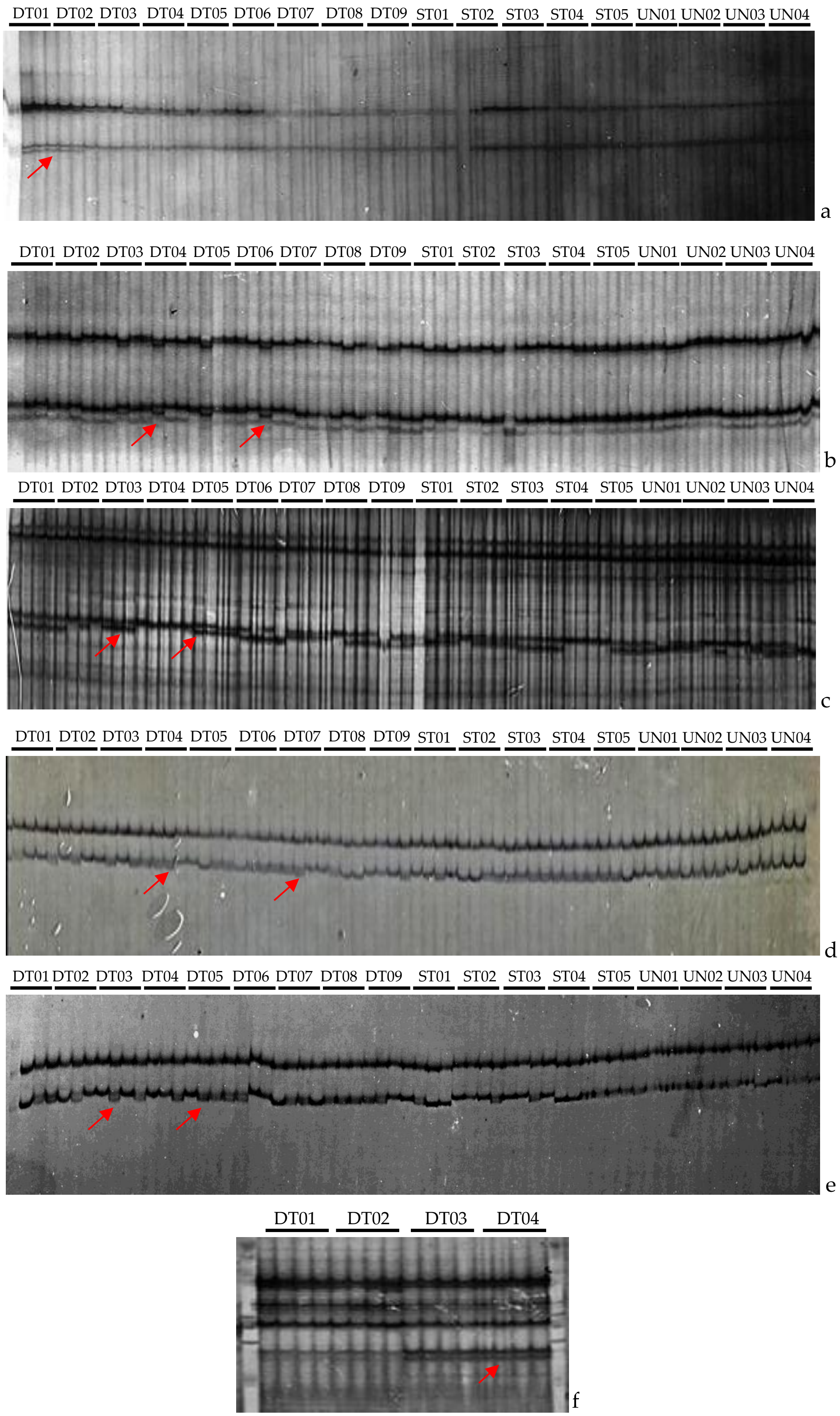

Figure S1. DNA fingerprints of oil palm sample examined with difference gene primers via SSCP in 29:1 nondenaturing polyacrylamide gel. (a) *cmo* (b) *ABA-04* (c) *MHAR-04* (d). *helicase-03* (e) *helicase-04* and (f) *P5CDH-01*. Solid arrows indicate different bands.

CGCTAAGCATGCATCTGGTCTTAGCTTGACTCCTACTCTACCCTTGTATGGACATTTCTTAATATGGTTT
TCTTATCTGTTATACATTCTCTGTGCATTGTTCTTGCTATAAATTTTCAACCTGGCATTGTAATATTGCA
GTGGAATGTCATCATCCTGTTTTTCATTCCATGTTTCTATTGTTTCTGAATCATGAATGCTATGATGGG
GAAACATGCACATTTTATGTAAGATTTGGCTTTCTTTCCAAACTGTGCTGTTCATCTTGCTTTGAAATC
GTACTTTGGTTCCACCTTCCTCCCAAATTATGTGGATCCATAGTTCACCACCAGCTTTGCTACCATATT
CCATATTCTCATCATGAGTATGTTAAAGTGATGTCATGCTTCTTAGCATATTTTTTCCCCTTTTCTTCGCT
GCATTTTCATGAGATGGAAGCTATAATACCTTTCTGGGACAAAATTTTCTTTTTCATTCTTTATCATGCT
GCCATGTAGATTTTTGAGAAGGTAAGTATTCAAAGATGTGAAGGTGCTTCTGTAGATGAAGTTGGCTT
TGATAGGCTGGGATCAAAAGCAGTGTATGCTTTCATTTATCCAAACTTCATGGTCAACAGGTGTGCTA
TTATTCAGTCCATAATTTCTCCTAATGAGGATCTGATGCTTATTCAATGAGTTATGGTAATTGATAGCT
CAGAGATTTGAGTTTTTCAAATGTTTACAGGTATGGTCCATGGATGGACACTATCTTA

a

CACCCGGGGTCGGGTGGCCGGACCCTCTACCTCTCCAATGTCAAAGTGGCCTTCTGCAGCGACCGCC
CTCTCTCCTTCACTGCCCCCTCAGGACAGCAGGCCTGGAGCTACTATAAGGTAAGACATGCTTCTTTT
GATCCACATTCATTTTAAAAGCATCAGCCGTTGTAGTTAAACTGGGAAAGTGGTTTACCTAAAGTTCA
TCGAGCATGATGAATGTTAATTTGATCATGTAGGTTGTGATCCCTCTAGCGTTGATTGCAACGGTTAAT
CCTGTGACAATGGCAGAGAATCCACCAGCTAAGA

b

TATGCCCAGGAAATCGCAGGCACGAGAATAGAAATATGGAAGGTAGTCATATTCCTCAATGTC
TTTTCCTTCTTCACTTGCCTTGATTGCCTGACAGCGTAGCAAAATCATAAACTTAGCAAACCAC
CGAATTTACATGCATTGCAGCCATTACAACTAATTGAAACTCAGTTACCTTAACAGCTTGTTCT
GCTGATTTTCTGGCATGATCAACATGCTCCACTCTCCTCATTTCATAGTACAGTTTCAAGGGGA
AGGTTGCTACATCTCCCACAGCATATACCCCAGGAACACTTGTTCTAAAGAATCCATCAGTCT
GCAACCAAAAAGGATAGATAAAGCTTTTTTTCACATCAAGACTAAAAACAATCACAACCAGT
GGCTAGATAGATTATAGGCCAACGCTTTCCATATATTGATATTTTTCATGTTTCTAATATATTTG
CTAGCTTCTTTCTGCCATTTTCATGTAAACATTTCTCAATGGATCACAAACTTCATGACAACAA
GAAGGAAAGACAGAAAGGGAAAGAAAAAGAAAGAAGCTTGTAAGGTAGACTCCAAAGGAGA
GAATTTTGACCTTGATTCCACCTTTCTCT

c

GCCAAAGAAGAATCAGTTAAAAGATACTATTGGTGATGCAGATGAGCCTGAAGGTACTTTATCCATT
TTAAAGTGCTTTTCTTAGTCAACAAAGGCATAATTGTAACTGACAATTGCAATTATCTAGAGTTGGGT
GATCCCTTGACTGTGGAGGAACAGGAAGAAAAGGAGCAGTTGTTAGAAGAGGTGCTACCACTTCTAG
ATCATTGAAAATCCTTCAGTCCCCTTAGTATCTGTTGATCTGACTGCTGCAAGTTTCAGGGTTTTTCAA
CATGGACAAGAAGAGATTTCAACACTTTTATCAGAGCATGTGAGAAGTATGGCCGGAATGACATAAA
AAGTATAGCTTCTGAAATGGAGGGCAAGACGGAGGAGGAAGTTGAGAGGATATGCCAAAAAC

d

GACAAGAAGGGAACAGGTTAAAGGATACTATTGGTGATGCAGATGAGCCTGAAGGTACTTTATCCAT
TTTAAAGTGCTTTTCTTAGTCAACAAAGGCATAATTGTAACTGACAATTGCAATTATCTAGAGTTGGGT
GATCCCTTGACTGTGGAGGAACAGGAAGAAAAGGAGCAGTTGTTAGAAGAGGTGCTACCACTTCTAG
ATCATTGAAAATCCTTCAGTCCCCTTAGTATCTGTTGATCTGACTGCTGCAAGTTTCAGGGTTTTTCAAC
ATGGACAAGAAGAGATTTCAACACTTTTATCAGAGCATGTGAGAAGTATGGCCGGAATGACATAAAA
AGTATAGCTTCTGAAATGGAGGGCAAGACGGAGGAGGAAGTTGAGAGATATGCCA

e

Figure S2. Nuclotide sequence of difference primers. (a) *cmo* (b) *ABA* (c) *MHAR* (d) *helicase-03* and (e) *helicase-04*.

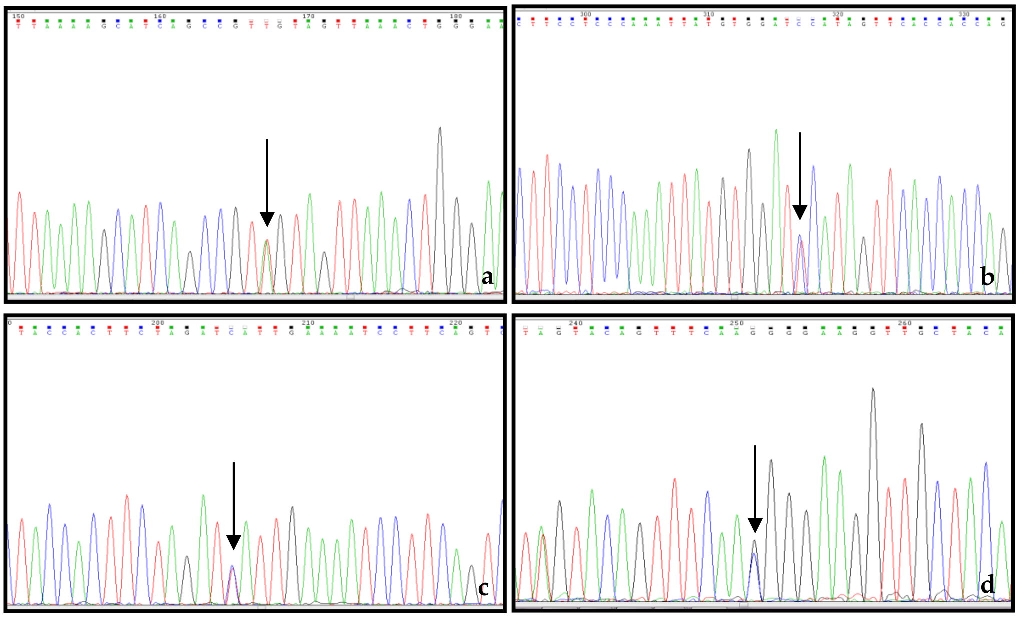

Figure S3. Chromatogram sequence revealed Single Nucleotide Polymorephisms (SNP position of various gene. (a) T/A SNP in *ABA-04* (b) C/T SNP in *cmo-03* (c) C/T SNP in *helicase-03* (d) G/C SNP in *MHAR-04*. Solid arrows indicate the positions of SNPs.

Table S1. Tetra primer of various genes designed from the SNP positions.

Primer name	Primer nucleotide	Number of base	PCR product (bp)
ABA-control-F	TGT CAA AGT GGC CTT CTG C	19	268
ABA-control-R	GGT GGA TTC TCT GCC ATT GT	20	
ABA-T-F	TTT TAA AAG CAT CAG CCC TT	20	159
ABA-A-R	CTT TCC CAG TTT AAC TTC T	19	147
CMO1-control-F1	GCA GTG GAA TGT CAT CAT CC	20	442
CMO1-control-R1	TGC TTT TGA TCC CAG CCT AT	20	
CMO1-C1-F	CCT CCC AAA TTA TGT GGT TC	20	282
CMO1-T1-R	GCT GGT GGT GAA CTA AGA	18	197
CMO2-control-F1	GCA GTG GAA TGT CAT CAT CC	20	442
CMO2-control-R1	TGC TTT TGA TCC CAG CCT AT	20	
CMO2-C2-F	TTT TTT CCC CTT TTC TTG GC	20	181
CMO2-T2-R	CCA TCT CAT GAA AAT GGA A	19	299
CMO3-control-F3	TCT CTG TGC ATT GTT CTT GC	20	536
CMO3-control-R3	GCA CAC CTG TTG ACC ATG A	19	
CMO3-T3-F	TAT GTA AGA TTT GGC TTT	18	398
CMO3-A3-R	GAA CAG CAC AGT TTG GAA TGT	21	176
HL3-control-F	GTG ATG CAG ATG AGC CTG AA	20	348
HL3-control-R	CAA CTT CCT CCT CCG TCT TG	20	
HL3-C-F	GGT GCT ACC ACT TCT AGT TC	20	197
HL3-T-R	GGA CTG AAG GAT TTT CAT TA	20	160
MHAR1-control-F1	GAT TGC CTG ACA GCG TAG C	19	329
MHAR1-control-R1	TAT GGA AAG CGT TGG CCT AT	20	
MHAR1-G-F	TTC ATA GTA CAG TTT CAA G	19	171
MHAR1-C-R	GAG ATG TAG CAA CCT TCG CG	20	186
MHAR2-control-F2	GAT TGC CTG ACA GCG TAG C	19	329
MHAR2-control-R2	TAT GGA AAG CGT TGG CCT AT	20	
MHAR2-T-F	CCA CTC TCC TCA TTT CTT T	19	194
MHAR2-A-R	CCT TCC CCT TGA AAC TGT TCT	21	174
MHAR3-control-F3	GAT TGC CTG ACA GCG TAG C	19	329
MHAR3-control-R3	TAT GGA AAG CGT TGG CCT AT	20	
MHAR3-T3-F	GTT TCA AGG GGA AGG TTC CT	20	170
MHAR3-A3-R	GTA TAT GCT GTG GGA GAT CTT	21	199

ABA: abscisic acid, *CMO*: choline monooxygenase, *Hl3*: helicase-3, *MHAR*: monohydroascorbate reductase

Table S2. Nucleotide sequence of primer using for molecular marker screening

Gene name	forward primer (F:5'--->3')	reverse primer (F:5'--->3')
CMO-01	CTN GGA AAT GTR GAA TTT G	GAT AAG GGC AYT GGA AGC
CMO-02	CTC CTY GCA TGT GGA AGT GGT C	CCC ARC CAT TCA TYT CCA AC
CMO-03	GGT GGR TAT CAT GTT CCA TAY G	GGG ACM RCT AGA TTA GTG T
CMO-04	CCT CCT CTC TCC GAT TCC C	GTG ATG CAT AAA TCC AAT CAG
CMO-05	TGT GAT GCA TAA ATC CAA TC	CAA ATA TGA GGC AAC GAG GTG
Arg-01	TAG CAG TCA TTC CAT CAA C	ATT GTC TTG ATC CTG CAT TC
Arg-02	CAG TCA TTC CAT CAA CAG TG	ATT GTC TTG ATC CTG CAT TC
GR-01	AGA AAG TGG AGG TAT GCT G	GTT GAA CCT AGA GGC TGT TG
GR-02	AGT GGA GGT ATG CTG AAC AC	GTT GAA CCT AGA GGC TGT TG
GR-03	AGA AAG TGG AGG TAT GCT G	GGT AGG AGT CCA AAC ACA AA
GR-04	AAA CCA GAC CAG AGA TTG TG	GGT GAT GTT ACA AAC CGA AT
GR-05	AAA CCA GAC CAG AGA TTG TG	GCA TTA ATG GAA GGA ACT TG
P5CDH-01	TTC GAT TCC ATA GAA GGA G	CTA TGA TAT TGG TCC CTT GC
P5CDH-02	ATA CAC ACG CAC ACA CTG AC	CTT GGG AAG TCT GTT AAT GG
BADH-01	TAA CCG AGA GGA AAT CTG AG	GCA TAT GTC AGC AAG CTC
BADH-02	ACA CGA CGA CTG AAC TAT G	GCA GTC ATG ACC TTC TTA C
MHAR-1	CTT TCT CCT CAA CAA CTT GG	GCT GCT TTC TAT GAG GGT TA
MHAR-2	ACA ATT TCC GTG CTG AGT ATG	GTG ATG GTC GAT TTG TTC
MHAR-3	CGG TAT CTC CAA CAT TGT C	CCA AGT TGT TGA GGA GAA AG
MHAR-4	ACG GTA TCT CCA ACA TTG TC	CAA GTT GTT GAG GAG AAA GG
MHAR-5	ACG GTA TCT CCA ACA TTG TC	CCA AGT TGT TGA GGA GAA AG
SOD-01	ACC AGA ATC CAT CAC TCA AG	CTC TGA AGG AGT TGC TGA
SOD-02	CTT GCT GAG CCA AAA GTA G	GAC ACT GGC AGA GTT GAA T
SOD-03	ATG AAC CAC AAA TGC TCT TC	GAC GAT GGT CCT ACA ACA GT
SOD-04	ACC AGA ATC CAT CAC TCA AG	GGC ACA TTT TAA CCC TAA CA
SOD-05	GTG ATC TTG GGA ATG TGA C	CAA ACT TAC CCT GAA AAT GC
APX-01	CAC GAC GAC TCC TTA TCA G	AAG CCT GAA CCC CCT AAG
APX-02	ACA CGA CGA CTC CTT ATC	AAG CCT GAA CCC CCT AAG
APX-03	ACG ACT GGC ATA TCT TTC C	CTT CAA GGA GCT TCT GAG TG
APX-04	GAC GAC TGG CAT ATC TTT CC	CTT CAA GGA GCT TCT GAG TG
TRE-01	TCC TGA TTT TGG ATC TTG TC	CCG ATA CAA AGA ACG AAG TC
TRE-02	TTA AAC ACC TTG TGT CGT G	GTT TCG GTG TAC AAC CTC
Helicase-01	AGG AAC AGG AAG AAA AGG AG	ATT ATC TCA TCC TTG CGA G
Helicase-02	AGG AAC AGG AAG AAA AGG AG	CCA ATT GCC TTC ATT ATC TC
Helicase-03	TGC CTT TTT GAT ATC TGT CC	TGG CAT ATC TCT CAA CTT CC
Helicase-04	GCC TTT TTG ATA TCT GTC C	TGG CAT ATC TCT CAA CTT CC

Table S2. Nucleotide sequence of primer using for molecular marker screening (Cont.).

**Gene name**	**forward primer (F:5'--->3')**	**reverse primer (F:5'--->3')**
catalase-01	GTC AGC ATA GGC AAA GAC TC	GGA AGC TCT ATG TTC AGG T
catalase-02	ACC TGA ACA TAG AGC TTC C	GAG CTT GCA CAC TTT CTT C
catalase-03	TTC ACA TAA TGG ACC TTT CC	TTT CCC AGT GTT CTT CAT TC
catalase-04	ACC TGA ACA TAG AGC TTC C	GAG CTT GCA CAC TTT CTT C
ABA-01	CTG GGA AAG TTT GAA GAC TG	CAC TTT GAC ATT GGA GAG G
ABA-02	GGA AAG TTT GAA GAC TGC AC	CAC TTT GAC ATT GGA GAG G
ABA-03	CTA ACG TCT GGG AAA GTT TG	CAC TTT GAC ATT GGA GAG G
ABA-04	AGA AGA CGT TTG CTT GCT AC	CTT AGC TGG TGG ATT CTC TG

CM: choline monooxygenase, *Ar:* arginase, *GR*: glutathione, *P5CDH*: pyrroline- 5- carboxylate dehydrogenase, *BAD:* betain aldehyde dehydrogenase, *MHAR*: monohydroascorbate reductase, *SOD*: superoxide dismutase, *APX*: ascorbate peroxidase, *ABA*: abscisic acid

Note : (Y) substitute with T, (CR) substitute with A, G, (N) substitute with A, T, C, G (M) substitute A, T, G
